# Environmental exposure to wildfire smoke may reduce microvascular oxygenation during graded handgrip exercise: A case series

**DOI:** 10.14814/phy2.16120

**Published:** 2024-06-20

**Authors:** Oliver E. Blum, Justin A. DeBlauw, Lauren M. Greaves, Elena S. Shostak, Stephen J. Ives

**Affiliations:** ^1^ Department of Health and Human Physiological Sciences Skidmore College Saratoga Springs New York USA

**Keywords:** air quality, cardiovascular, health, particulate matter, wildfire

## Abstract

Wildfire smoke (WFS) is an urgent and rapidly growing threat to global health. Aside from obvious threats to pulmonary function, increases in cardiac abnormalities or myocardial infarction have been documented during WF season, but little is known about the effects of WFS on cardiovascular health. We investigated the effect of nonoccupational WFS exposure on cardiovascular and pulmonary function at rest and during graded handgrip exercise through a case series of young, healthy adults (*n* = 4, 25 ± 6 years) assessed after ≥3 days of bad or good air quality. Peripheral and estimated central blood pressures, vascular stiffness, and microvascular function (Near infrared spectroscopy, NIRS) were assessed at rest, and during rhythmic handgrip exercise. WFS did not appear to alter resting peripheral, central BP, or vascular stiffness (all, *p* > 0.05). Slope 1 and slope 2 from the NIRS‐vascular occlusion test (NIRS‐VOT) were not different between conditions (*p* > 0.05). The change in SmO_2_ during exercise was lower (*p* = 0.02, $\eta_{p}^{2}$ = 0.62) with bad air quality. These preliminary findings suggest modest effects of environmental WFS exposure on muscle microvascular function during exercise in healthy adults. Future work is needed to elucidate the physiological changes with WFS exposure and the increased risk of cardiovascular events, perhaps exacerbated through physical activity.

## 1 INTRODUCTION

Wildfire smoke (WFS) is an emerging threat to global health. Particulate matter (PM) is a principal component of WFS, and PM smaller than 2.5 micrometers in diameter (PM_2.5_) is of particular interest for its ability to reach the bloodstream (Naserinejad et al., 2023). Epidemiological evidence has linked WFS to increased pulmonary mortality/morbidity, but it has also been documented to increase cardiovascular events and mortality (Gao et al., 2023).

Pathophysiological changes in the cardiovascular system resulting from PM_2.5_ exposure (e.g., exhaust) include increased augmentation index (AIx) and pulse wave velocity, decreased reactive hyperemia index, and altered diameter of retina microvasculature (Li et al., 2023; Naserinejad et al., 2023; Zhang et al., 2016). These findings would suggest vascular stiffening and impairment of microvascular function. Indeed, reductionist studies reveal oxidative stress and endothelial cell dysfunction with WFS/PM_2.5_ exposure (Black et al., 2017; Chen et al., 2021). However, these mixed sources of PM_2.5_ are different in composition from WFS, and there is scant evidence of the latter's effect on cardiovascular function. A review of controlled wood smoke exposure studies highlighted conflicting effects on cardiovascular function (Chen et al., 2021), thus more work is needed to elucidate the effects of WFS on factors related to cardiovascular risk (e.g., vascular function).

To date, no study has assessed the effect of nonoccupational environmental WFS exposure on cardiovascular health and microvascular function at rest and during exercise. Thus, we investigated the effect of environmental WFS exposure on cardiovascular and microvascular function at rest and during exercise in a case series.

## 2 METHODS

### 2.1 Participants and general procedures

Data were obtained from four healthy adults (three males, one female) from the Saratoga Springs, NY area. Healthy was defined as individuals who were non‐smokers and free of cardiovascular, pulmonary, metabolic, musculoskeletal, neural, metastatic, or renal disease. This case series was executed through a retrospective review of de‐identified data and thus was considered exempt by the local Institutional Review Board (IRB#2402–1142), and was conducted in accordance with the CARE guidelines, as applicable.

### 2.2 Protocol

To understand the potential cardiovascular implications of exposure to WFS, and particulate matter (primarily PM_2.5_), associated with the Canadian wildfires in Quebec Province, the current study utilized a retrospective case series. Analyzing existing deidentified data, and dates corresponding with air quality values, individuals were assessed on two separate occasions: after ≥3 consecutive days of poor air quality and after three cumulative days of good air quality, which was 6 days after the initial assessment. Bad air quality was determined as being >150 AQI for PM_2.5_ (US air quality index per https://www.airnow.gov/), and the AQI averaged 166. Good air quality was determined as being <50 AQI for PM_2.5_, which averaged 49. Data were collected at approximately the same time of day (15:00 h). The same procedures, and their order, were performed for each visit.

Upon arrival, participants rested for 5 min in a temperate, quiet, dimly lit room in a semirecumbent position. First, heart rate, peripheral blood pressure, estimated central blood pressure, and pulse wave analysis (augmentation index, AIx) were measured using the automated oscillometric blood pressure cuff technique (Sphygmocor Xcel; Atcor, Naperville, IL, USA) (Alpert et al., 2014) using standard methods as described previously (Matias et al., 2023; Schlicht et al., 2018). Briefly, central (aortic) BP were derived from the peripheral pressure waveform at the brachial artery using a general transfer function. Afterwards, a 5‐min near‐infrared spectroscopy (NIRS) vascular occlusion test (VOT) was conducted to assess resting microvascular function of the forearm, using standard methods as described previously (Mclay et al., 2016; Zaleski et al., 2023), using a NIRS probe (Moxy, Fortiori Design LLC, Hutchinson, MN, USA). The desaturation rate was calculated as the slope of the first 10 s upon occlusion (Azevedo et al., 2022; Greaves et al., 2024), while the reperfusion rate was calculated in the first 10 s post cuff‐release (Mclay et al., 2016), as markers of muscle metabolism and vascular reactivity, respectively.

After a brief rest, a maximal voluntary contraction using a handgrip dynamometer (Jamar, Warrenville, IL, USA) was performed. The participants then performed a graded rhythmic (1 Hz) handgrip protocol with 3‐min stages of 2 kg, 4 kg, and 8 kg resistance. Halfway through each stage (90 s), BP was recorded to gain insight into possible WFS‐induced alterations in the exercise pressor reflex (EPR).

Finally, participants underwent an assessment of dynamic lung function at rest using a handheld digital spirometer (MicroPlus Spirometer, CareFusion, Chatham, UK). Pulmonary function testing for forced expiratory volume over 1 s (FEV_1_), forced vital capacity (FVC), and FEV_1_/FVC ratio were assessed using standard technique, as described previously (Giuriato et al., 2020).

### 2.3 Statistical analysis

Statistical analysis and figures were generated using open‐source software (JASP, v.0.16.3, University of Amsterdam, Amsterdam, NED). Paired t‐tests or two‐way repeated measures ANOVA were used to assess the impact of air quality on function, as appropriate. Tests of normality were performed and if a violation was found, an alternative procedure was performed. As a goal of this study is to provide effect sizes for future research, estimates of effect size were determined by Cohen's *d* and partial eta squared ($\eta_{p}^{2}$) using conventions to indicate small, medium, and large effects within each. Alpha was set at 0.05. Data are mean ± SD, unless otherwise stated.

## 3 RESULTS

### 3.1 Participant characteristics and effect of WFS on pulmonary function

Participants were young (24.8 ± 6 years), on average 177.8 ± 8 cm tall, 79.8 ± 13 kg in mass, and normal body weight (BMI 25.1 ± 1.9 kg/m^2^). FEV_1_ was not affected by WFS (*p* = 0.899, *d* = 0.083). FVC was lower during good air quality compared to bad air quality (4.327 L vs. 4.463 L, respectively, *p* = 0.03, *d* = 3.283). FEV_1_/FVC was not affected by WFS (*p* = 0.502, *d* = −0.469).

### 3.2 Effect of WFS on resting blood pressure, vascular stiffness, and microvascular function

**FIGURE 1:**
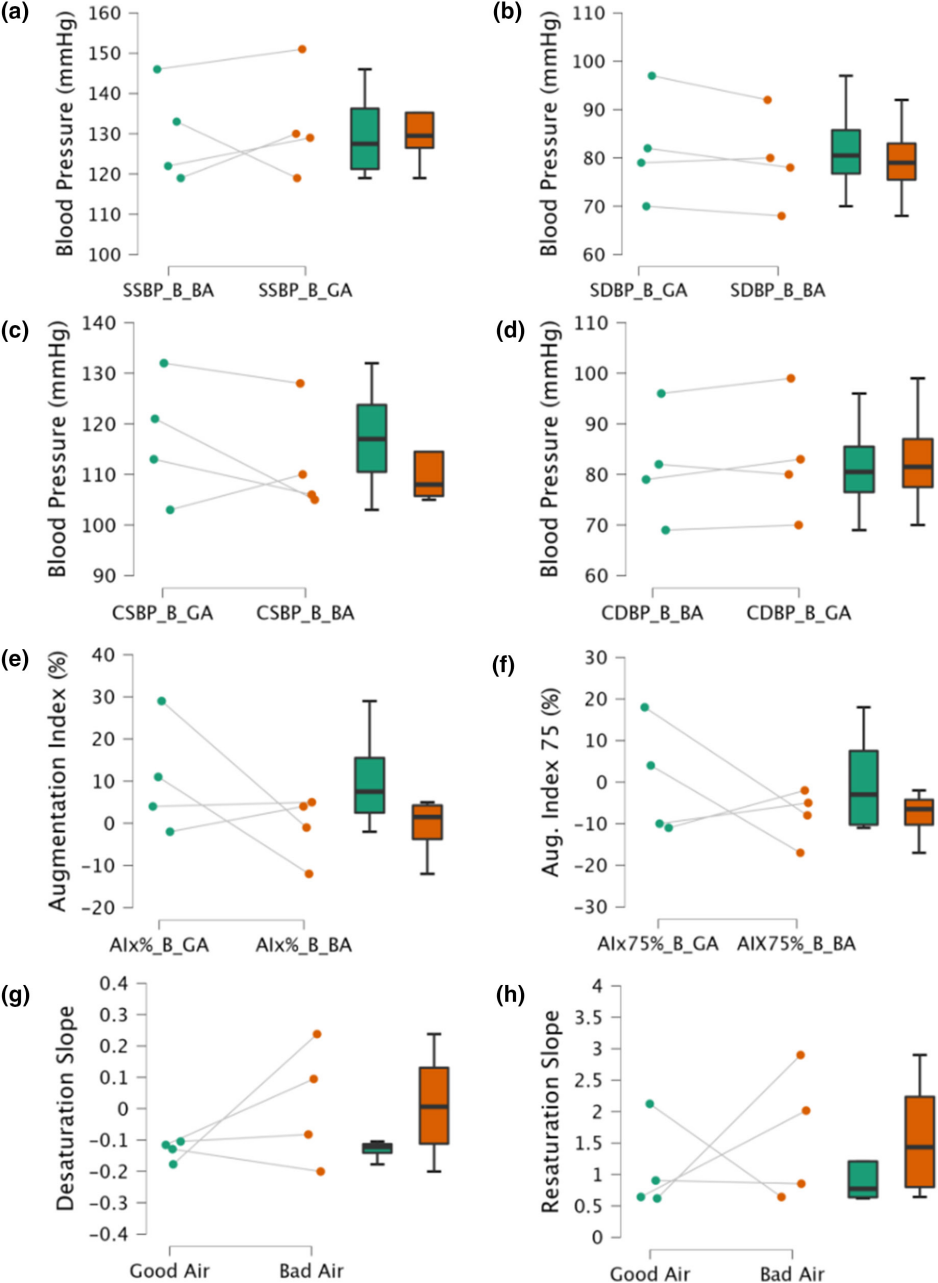
Cardiovascular indices after ≥3 days of environmental wildfire smoke exposure (BA) and after three cumulative days of good air quality (GA). (a) Peripheral brachial artery systolic blood pressure. (b) Peripheral brachial artery diastolic blood pressure. (c) Central systolic blood pressure. (d) Central diastolic blood pressure. (e) Augmentation index (AIx%). (f) Augmentation index normalized to 75 bpm (AIx 75%). (g) Desaturation slope (slope 1) during NIRS VOT. (h) Resaturation slope (slope 2) during NIRS VOT. Paired samples *t*‐tests were used to compare the good air quality (GA) and bad air quality (BA) on cardiovascular indices. Values are mean ± 95% confidence interval. *N* = 4.

Resting peripheral systolic (PSP) and diastolic (PDP) BP were not affected by WFS (*p* = 0.713, *d* = 0.202; *p* = 0.155, *d* = 0.948, respectively, Figure 1a,b). Central systolic (CSP) and diastolic (CDP) BP were not affected by WFS (*p* = 0.369, *d* = −0.527; *p* = 0.339, *d* = −0.567, respectively, Figure 1c,d). Mean arterial pressure (MAP) and pulse pressure (PP) were not affected by WFS (*p* = 0.48, *d* = −0.403; *p* = 0.165, *d* = −0.913, respectively, data not shown). AIx and AIx75 were not affected by WFS (*p* = 0.284, *d* = −0.651; *p* = 0.422, *d* = −0.463, respectively, Figure 1e,f). The desaturation rate (*p* = 0.273, *d* = 0.669) and reperfusion rate (*p* = 0.566, *d* = 0.322) during the NIRS‐VOT were also not affected by WFS (Figure 1g,h).

### 3.3 Effect of WFS on pressor response to handgrip exercise

There was no effect of WFS or stage on PSP during exercise (*p* = 0.502, $\eta_{p}^{2}$ = 0.162; *p* = 0.224, $\eta_{p}^{2}$ = 0.370, respectively), nor an interaction effect (*p* = 0.387, $\eta_{p}^{2}$ = 0.274). There was no interaction of WFS and stage on PDP during exercise (*p* = 0.642, $\eta_{p}^{2}$ = 0.097) or of WFS on PDP during exercise (*p* = 0.743, $\eta_{p}^{2}$ = 0.041). However, there was an effect of stage on PDP during exercise (*p* = 0.048, $\eta_{p}^{2}$ = 0.568). There was no effect of WFS or stage on CSP during exercise (*p* = 0.882, $\eta_{p}^{2}$ = 0.009; *p* = 0.066, $\eta_{p}^{2}$ = 0.533, respectively), and there was no interaction effect (*p* = 0.217, $\eta_{p}^{2}$ = 0.375). There was no effect of WFS on CDP during exercise (*p* = 0.972, $\eta_{p}^{2}$ < 0.01). However, there was a large effect of stage on CDP during exercise (*p* = 0.038, $\eta_{p}^{2}$ = 0.59). There was no interaction of WFS and stage on CDP (*p* = 0.922, $\eta_{p}^{2}$ = 0.05). There was no effect of WFS or stage on MAP during exercise (*p* = 0.868, $\eta_{p}^{2}$ = 0.011; *p* = 0.178, $\eta_{p}^{2}$ = 0.441, respectively), and there was no interaction effect (*p* = 0.844, $\eta_{p}^{2}$ = 0.083).

### 3.4 Effect of WFS on microvascular response to handgrip exercise

**FIGURE 2:**
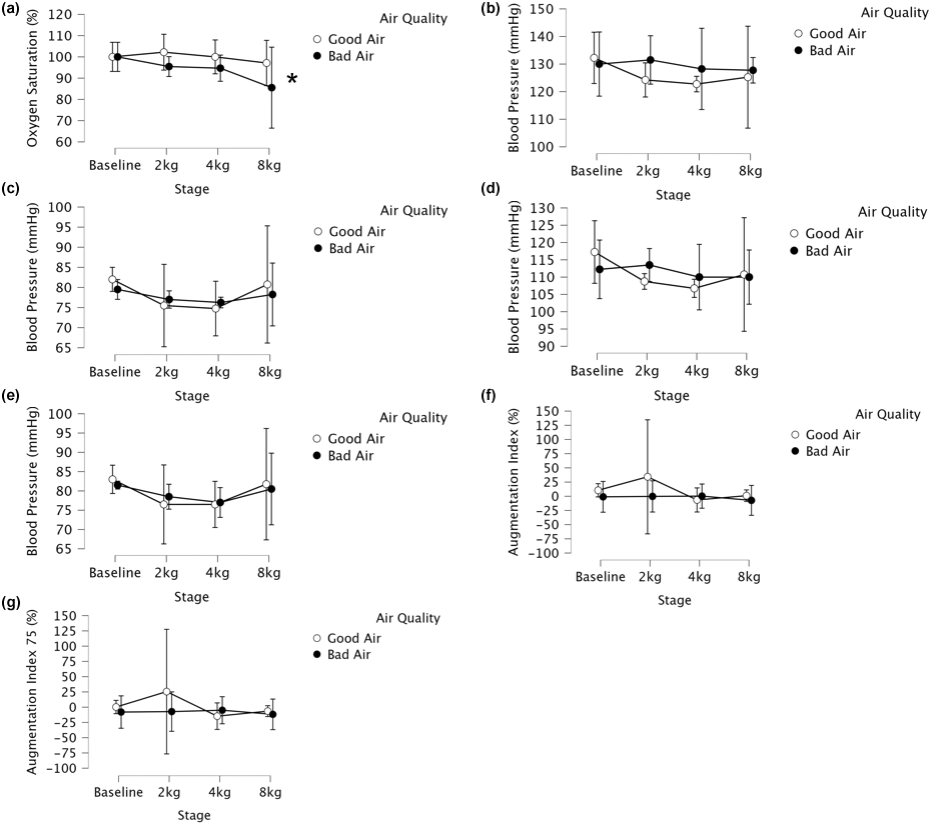
Forearm muscle microvascular oxygenation (SmO_2_%) and cardiovascular indices during handgrip exercise after ≥3 days of environmental wildfire smoke exposure (bad air) and after three cumulative days of good air quality (good air). (a) Forearm muscle microvascular oxygenation (SmO_2_%, % of initial) (b) Peripheral Brachial (B) Artery systolic blood pressure. (c) Peripheral Brachial (B) Artery diastolic blood pressure. (d) Central systolic blood pressure. (e) Central diastolic blood pressure. (f) Augmentation index (AIx%). (g) Augmentation index normalized to 75 bpm (AIx 75%). Two‐way repeated measures ANOVA were used to compare the effect of bad air and good air quality on cardiovascular indices and oxygen saturation over time. *significant interaction effect. Values are mean ± 95% confidence interval. *N* = 4.

WFS exposure tended to decrease SmO_2_ to a greater extent during exercise that trended toward significance (*p* = 0.146, $\eta_{p}^{2}$ = 0.559), and there was a significant effect of stage (*p* = 0.043, $\eta_{p}^{2}$ = 0.578). There was no interaction of WFS and stage on SmO_2_ during exercise (*p* = 0.327, $\eta_{p}^{2}$ = 0.306). There was no effect of WFS or stage on THb during exercise (*p* = 0.624, $\eta_{p}^{2}$ = 0.09; *p* = 0.825, $\eta_{p}^{2}$ = 0.091, respectively), and there was no interaction (*p* = 0.475, $\eta_{p}^{2}$ = 0.232). However, when the SmO_2_ data were normalized to baseline there was an interaction of air quality and exercise intensity (*p* = 0.02, $\eta_{p}^{2}$ = 0.62) where SmO_2_ was attenuated more in the bad air quality (Figure 2).

## 4 DISCUSSION

This may be the first study to assess the effect of nonoccupational environmental WFS exposure on cardiovascular and microvascular function at rest and during exercise. WFS exposure did not affect central or peripheral BP or vascular stiffness at rest in this case series of young healthy individuals. Resting muscle metabolism and microvascular function were not affected by WFS exposure. However, muscle microvascular oxygenation was lower during exercise with WFS exposure, suggesting that it may impair microvascular function during exercise. A decrease in microvascular function, as seen with other PM_2.5_ sources, could be a pathophysiological consequence of WFS exposure, bridging the well‐documented association between WFS exposure and cardiovascular events, with physical activity as a revealing factor.

Regarding lung function, WFS exposure did not affect FEV_1_ or FEV1/FVC compared to good air quality. Surprisingly, FVC was lower during good air quality compared to poor air quality. Given that participants were exposed to WFS first, it is possible that WFS had a delayed and residual effect upon FVC. A study of Montana residents exposed to WFS during the summer of 2017 found that their FEV_1_/FVC was comparable to predicted values at the end of the summer but then decreased and remained depressed 1 and 2 years later, respectively (Orr et al., 2020). Although the Montana residents were cumulatively exposed to far more WFS PM_2.5_, further study is needed in this area, including out of hospital assessments of the lay population, and in areas not typically exposed to WFS.

In the present study, WFS exposure did not affect peripheral and central blood pressure at rest. Although no other study has investigated the effect of WFS on blood pressure or show dubious findings (Chen et al., 2021), assessing central and peripheral pressure is warranted, while they are related they can show independent findings and central pressures are highly predictive. Regarding vascular stiffness, we did not observe changes in mean arterial pressure, pulse pressure, or augmentation indexes. However, studies of wildfire firefighters, wood cookstoves, and controlled wood smoke exposure have observed increases in Alx (Chen et al., 2021). We found no effect of WFS on the reperfusion rate with reactive hyperemia. Although no other study has assessed the reperfusion rate after nonoccupational WFS exposure, a study of controlled wood smoke exposure, up to 354 μg/m3 for 3 h, also found no effect on reactive hyperemia (Forchhammer et al., 2012).

In the current study, WFS exposure did not alter the EPR in response to graded handgrip exercise. Perhaps greater muscle mass and/or intensities are needed, or in more vulnerable populations, to reveal any difference in exercise pressor response that WFS may have induced, but to date, no other study has investigated EPR in response to WFS or PM_2.5_. We did observe lower microvascular oxygenation during exercise after bad air quality. After bad air quality, there was a mean difference in muscle oxygen saturation of −14% during 8 kg resistance compared to baseline, versus only −3% after good air quality. No other study, that we are aware of, has assessed the effect of WFS on muscle microvascular oxygenation. However, a single 3‐year study of rice‐crop residue burning in the Indian state of Punjab found no change in resting oxygen saturation at the finger before, during, or after the burning seasons (Agarwal et al., 2012). Aside from many differences in methodology including tissue site, our use of exercise stress, and measurement technique, our difference in findings may be due in part to PM_2.5_ concentrations relative to other pollutants. Unlike the present study, the pollutant of greatest concentration was not PM_2.5_ in the study of Agarwal and colleagues (Agarwal et al., 2012). Participants' time outdoors and thus WFS exposure was not documented, which may be dose dependent for cardiovascular outcomes (Chen et al., 2021), or may be sex dependent (Greaves et al., 2024) and thus should be considered in the future.

### 4.1 Conclusion

In this case series, we explored the effect of nonoccupational environmental exposure to WFS on cardiovascular and microvascular function at rest and during exercise in healthy adults, revealing decreased microvascular oxygenation during exercise with WFS exposure. Microvascular dysfunction during exercise due to WFS could help to elucidate the association between exposure and cardiovascular events and the potential complication of physical activity.

## FUNDING INFORMATION

The American Heart Association has provided support to SJI (https://doi.org/10.58275/AHA.24AIREA1247045.pc.gr.189804).

## CONFLICT OF INTEREST STATEMENT

The authors have no potential conflicts of interest to disclose.

## ETHICS STATEMENT

This case series review of retrospective analysis of deidentified records was reviewed and approved under exempt status by the Skidmore College IRB (#2402–1142).

## Data Availability

The data that support the findings of this study are available from the corresponding author upon reasonable request.
